# Exploring Dimensionality Reduction Techniques in Multilingual Transformers

**DOI:** 10.1007/s12559-022-10066-8

**Published:** 2022-10-29

**Authors:** Álvaro Huertas-García, Alejandro Martín, Javier Huertas-Tato, David Camacho

**Affiliations:** Departamento de Sistemas Informáticos, Universidad Politécnica de Madrid, Madrid, Spain

**Keywords:** Dimensionality reduction, Natural language processing, Semantic textual similarity, Multilingual transformers, Language models

## Abstract

In scientific literature and industry, semantic and context-aware Natural Language Processing-based solutions have been gaining importance in recent years. The possibilities and performance shown by these models when dealing with complex Human Language Understanding tasks are unquestionable, from conversational agents to the fight against disinformation in social networks. In addition, considerable attention is also being paid to developing multilingual models to tackle the language bottleneck. An increase in size has accompanied the growing need to provide more complex models implementing all these features without being conservative in the number of dimensions required. This paper aims to provide a comprehensive account of the impact of a wide variety of dimensional reduction techniques on the performance of different state-of-the-art multilingual siamese transformers, including unsupervised dimensional reduction techniques such as linear and nonlinear feature extraction, feature selection, and manifold techniques. In order to evaluate the effects of these techniques, we considered the multilingual extended version of Semantic Textual Similarity Benchmark (mSTSb) and two different baseline approaches, one using the embeddings from the pre-trained version of five models and another using their fine-tuned STS version. The results evidence that it is possible to achieve an average reduction of $91.58\% \pm 2.59\%$ in the number of dimensions of embeddings from pre-trained models requiring a fitting time $96.68\% \pm 0.68\%$ faster than the fine-tuning process. Besides, we achieve $54.65\% \pm 32.20\%$ dimensionality reduction in embeddings from fine-tuned models. The results of this study will significantly contribute to the understanding of how different tuning approaches affect performance on semantic-aware tasks and how dimensional reduction techniques deal with the high-dimensional embeddings computed for the STS task and their potential for other highly demanding NLP tasks.

## Introduction

Natural Language Processing (NLP) includes various disciplines that provide a system with the ability to process and interpret natural language, just like humans use language as a communication and reasoning tool [1]. Due to the recent increases in computational power, parallelisation, the availability of large data sets, and recent advances in Artificial Intelligence, especially in the Machine Learning research field, NLP has been steadily proliferating and has garnered immense interest [1, 2]. In recent years, transformer-based architectures [3] have become an indispensable staple in the NLP field. Transformer models can capture latent syntactic-semantic information and encode text’s meaning as contextual vectors in a high-dimensional space referred to as embeddings [2, 4]. In contrast to previous approaches, such as Statistical Natural Language Processing, using the attention mechanism provided by these architectures allows us to consider many characteristics involved in human language.

The tremendous power of transformer-based models in Natural Language Understanding (NLU) and the new models that are continuously being proposed allows us to improve the state-of-the-art results in varied NLP tasks dramatically, including question answering, sentence classification, and sentence-pair regression like Semantic Textual Similarity (STS) [2, 4–6]. The semantic evaluation performed in this STS task is one of the “levels of language” that determines the possible meanings of a sentence by focusing on the interactions among word-level [7]. Given the many different linguistic features involved, it entails a high degree of complexity. In STS tasks, the systems must compute how similar two sentences are considering all these features, returning a similarity score, usually ranging between 0 and 5 [8].

Regarding sentence-pair regression tasks, to overcome the massive computational overhead caused by the quadratic dependence on the input size of the attention mechanism in transformers models [5, 9], the use of siamese architectures is a very effective method for efficiently deriving semantically meaningful sentence embeddings [5, 9]. This approach is also called dual-encoder, bi-encoder or siamese architectures. As explained by Humeau et al. [9], the training of a siamese architecture consists of two pre-trained transformer-based models with tied weights that can be fine-tuned for a specific task like computing separately semantic embeddings for a pair of sentences and measure their similarity using the extensively used cosine similarity function [10].

Despite the practical features of cosine similarity, such as symmetry and spatial interpretation, this similarity metric has complexity *O*(*N*): time and memory grow linearly with the number of dimensions of the vectors compared [11]. Thus, dimensionality is a bottleneck for similarity computation and embedding storage [12]. Moreover, an increasing number of studies using ensemble approaches based on the concatenation of embeddings can be found in the literature [13–15], aiming to improve the results in state-of-the-art tasks but accentuating this issue. Given that the application of dimensionality reduction techniques can mitigate this bottleneck, it requires further exploration.

Although in the history of NLP, the focus has mainly been on proposing architectures for English tasks. Nevertheless, interest in developing multilingual NLP tools has grown recently to achieve diversity for transferring NLP capabilities to low-resource languages for which labelled (and unlabelled) data is scarce [16, 17]. The incorporation of tasks in various languages in the SemEval [18] and CLEF [19] competitions is clear evidence of this, surmounting the language bottleneck, but also the increasing number of newly introduced multilingual models [19], such as the recent BLOOM (BigScience Language Open-science Open-access Multilingual) [20] open-sourced model with 176B parameters generated by BigScience which has marked an inflexion point on the research and development of large language models (LLMs).

The present paper seeks to address how different dimensionality reduction techniques impact the performance of pre-computed embeddings from multilingual transformer-based models trained using a siamese architecture focusing on semantic similarity by employing four different approaches where the reduction techniques are compared with the pre-trained and fine-tuned versions of the models.

This research aims to expand the current knowledge further on the effect of dimensionality reduction techniques in Natural Language Processing [21–24]. The following are the new contributions of our research concerning previous studies:

- A more comprehensive range of unsupervised dimension reduction techniques is included, from linear and nonlinear feature extraction to feature selection and manifold techniques.
- The effect of these techniques on pre-trained and fine-tuned pre-computed embeddings in the Semantic Textual Similarity (STS) task is explored.
- In contrast to previous work that explored reduction techniques in classical static word embeddings, this paper investigates the effect of dimension reduction in state-of-the-art contextual-based transformer models.
- Unlike previous works focused on the English language, this research analyses multilingual models, overcoming the language bottleneck for the applicability of dimension reduction of the embeddings of these models.

The remainder of the article is organised as follows: the “Related Work’’ section outlines previous work on dimensionality reduction techniques, their application in Deep Learning, and the importance of multilingual semantics in NLP. The “Methodology’’ section describes the approaches followed in this research to evaluate the impact of dimensionality reduction techniques, the multilingual models and the dimensionality reduction techniques applied, and the “Experimental Setup’’ section the data and processes used to fit and evaluate these techniques. The experimental results are discussed in the “Results’’ section. Finally, the “Conclusion’’ section summarises the results of this work and concludes.

## Related Work

### Dimensionality Reduction Techniques

Dimensionality reduction techniques aim to reduce the dimensionality of the data, removing irrelevant and redundant features while preserving critical information for subsequent applications, such as facilitating their visualisation and understanding or leading to more compact models with better generalisation ability [25–27].

There are different non-mutual exclusive criteria to classify dimensionality reduction techniques. Firstly, according to the reduction approach, these techniques can be classified into feature selection and feature extraction techniques [28]. Feature selection involves selecting a subset of original features useful for building models, effectively removing some of the less relevant features from consideration [26, 29]. On the other hand, feature extraction transforms the original data into another feature space with specific criteria, creating new variables by combining information from the original variables that capture much of the information contained in those original variables [25, 28]. Additionally, feature extraction methods can be further subdivided into linear and nonlinear according to the variable combinations applied [30].

Secondly, according to the information available in the datasets, dimensionality reduction techniques can be classified as supervised and unsupervised [25, 26]. Supervised techniques require each data instance in the dataset to be labelled accordingly to the task, whereas unsupervised techniques are task agnostic approaches and do not require labelled data.

### Dimensional Reduction of Embeddings

Broadly, embeddings can be categorised as pre-trained or downstream fine-tuned embeddings [31], and pre-computed or on-the-fly embeddings [32, 33].

The first criterion to categorise embeddings is whether they come from pre-trained models for general tasks or are task-specific. Pre-trained embeddings are widely used as a starting point for downstream applications. A clear example is transformer models’ current ‘Pre-train and Fine-tune’ paradigm [31]. Training these models from scratch is prohibitively expensive in many cases. Alternatively, using self-supervised trained checkpoints of these models and their pre-trained embeddings as a starting point to be later fine-tuned for supervised downstream tasks is widely used. Unlike previous works in the literature that have only focused on reduced pre-trained embeddings [21–23], in this work, we are interested in evaluating the impact of dimensionality reduction on both types of embeddings, pre-trained and downstream fine-tuned embeddings.

Pre-computed embeddings in NLP are widespread, i.e. embeddings that may or may not be adjusted to a task but are generated beforehand and not at each use time. A straightforward application of pre-computed embeddings is the semantic search NLP task for locating relevant information from massive amounts of text data [34]. A semantic search task uses semantic textual similarity to compare an input text against a large set of texts from a database to extract relevant related information-typically, a distance metric such as cosine similarity ranks which content should be extracted to an input query. However, computing the database embeddings each time a query is introduced is infeasible. Alternatively, it is preferable to compute the embeddings once, store them, and use these pre-computed embeddings for subsequent requests [33]. With this in mind, it is essential to note the usefulness of reducing embedding dimensions, which can improve their utility in memory-constrained devices and benefit several real-world applications.

As Camastra and Vinciarelli mention [35], using more features than is strictly necessary leads to several problems, pointing out that one of the main problems was the space needed to store the data. As the amount of available information increases, the compression for storage becomes even more critical [12, 36, 37]. Additionally, for the scope of this work, it cannot be ignored that the application of dimensional reduction techniques for reducing pre-computed embedding dimensions neither improves the runtime nor the memory requirement for running the models. It only diminishes the needed space to store embeddings. Besides, it increases the speed of making computations (i.e. to calculate the cosine similarity between two vectors), which also contributes to decreasing the considerable impact on the energy and carbon footprints generated during the production use of the models when pre-computed and stored embeddings are required [38]. Research has tended to focus on implementing bigger and more complex models rather than analysing methods to adjust the vector space to the desired task while conserving the required dimensions [38]. The additional problem is that the storage of high-dimensional embeddings is challenging when dealing with large volume datasets [33]; besides, researchers have already raised awareness of the storage limitation we are about to face if current technology is adopted and the storage utilisation growth rate persists [12].

In terms of performance, dimensionality reduction techniques can also contribute. The performance of Machine Learning (ML) and, in particular, Deep Learning (DL) models is highly dependent on the choice of data representation (or features) to which they are applied [39]. For that reason, much effort during the deployment of ML and DL solutions is dedicated to obtaining a data representation that can support effective learning. As a result, different fields where dimension reduction techniques are combined with ML and DL complex models can be found in the literature. For example, from their application in time series forecasting [40] or health sciences for cancer classification [41] to Natural Language Processing (NLP) to improve the clustering of text documents [42] or sentiment classification of opinion texts [17].

Recently, the study of Raunak et al. [21, 22] has shed more light on the importance of reducing the size of embeddings produced by ML and DL models in the field of NLP. More specifically, these authors focus on reducing the size of classical GloVe [43] and FastText [44] pre-trained word embeddings using PCA-based post-processing algorithms, achieving similar or even better performance than the original embeddings.

Other works on the potential of reducing pre-computed embeddings dimensions have been carried out [23], exploring the effect of Principal Components Analysis (PCA) [45, 46] and Latent Semantic Analysis (LSA) [24] dimensionality reduction techniques as a post-processing step of pre-trained GloVe word embeddings for text classification tasks. These authors also corroborated the usefulness of PCA for obtaining more accurate results with lower computational costs concluding that the PCA method is more suitable than LSA for dimensionality reduction. In the same way, Shimomoto et al. [47] propose solving topic classification and sentiment analysis by using PCA to transform pre-computed word embeddings of a text into a linear subspace. Their results showed the effectiveness of using the PCA subspace representation for text classification. Other authors have already proved this fact [48], showing that the storage, memory, and computation required by these large embeddings typically results in low efficiency in NLP tasks, pointing out the importance of methods to compress pre-computed and pre-trained GloVe and FastText embeddings.

Additionally, researchers have explored dimensional reduction techniques for visualising semantic embeddings of small text corpora [49]. The authors explored four dimension reduction strategies on pre-computed embeddings based on PCA and t-distributed stochastic neighbour embedding (t-SNE) [50], concluding that both methods preserved a significant amount of semantic information in the full embedding.

Similarly, the use of dimension reduction techniques is likewise interesting in Semantic Similarity [27, 37]. As discussed previously, in the Semantic Similarity task, the linear *O*(*N*) complexity of cosine similarity is one of the reasons why this distance metric is widely used in the community and this study. However, the complexity of cosine similarity, although linear, is a limitation when dealing with many dimensions and a large amount of data. Precisely, in tasks where cosine similarity is used as a distance metric, the large amount of data handled and the high number of dimensions of the embeddings generated by the models used represent a constraint in both computational time and storage that leaves the door open to the use of dimension reduction techniques.

To the best of our knowledge, in the literature, dimension reduction research on embeddings has focused on statistical methods, such as Bag of Words and Term Frequency-Inverse Document Frequency (TF-IDF) [27, 37], and classical pre-computed word embeddings, including the popular GloVe or FastText embeddings [21–24, 36, 49]. These classical word embeddings are more complex and powerful than statistical methods. However, they are static, word-level, and contextual independent, and their main limitation is that they do not consider what context the word is being used. Moreover, the varied embedding techniques explored are limited, focusing mainly on PCA. Nevertheless, it should be mentioned that novel dimension reduction approaches based on this classical technique [27] and feature selection methods [37] are being proposed. In fact this proves the relevance and importance of further exploring embedding dimension reduction. Likewise, these studies do not include multilingualism in their analyses, being limited to the English language.

Hence, the presented study follows the research line proposed by different authors [21–24] but takes a step forward, including a broader range of techniques and evaluating the capability of **dimensionality reduction techniques** in both **pre-trained and fine-tuned pre-computed embeddings** from state-of-the-art contextual-based transformer models from the recently claimed **multilingual** point of view.

### Siamese and Non-Siamese Architectures

Training in non-siamese transformer architectures, known as cross encoders, requires that two sentences be passed to the transformer as input while a target value is predicted [9]. This approach requires feeding the model with both sentences, which causes massive computational overhead because the attention is quadratic to the sequence length. As previously described in the “Introduction’’ section, siamese architectures are the main alternative to cope with this massive computational overhead in transformers models. As Reimers and Gurevych [5] measured, in a V100 GPU, finding the pair with the highest similarity in a collection of $n=10000$ sentences for a non-siamese model requires about 65 h. On the other hand, the same task with a model trained with a siamese architecture is reduced to $\sim 5$ s.

Although it should also be noted that models trained in a non-siamese way generally obtain better results [51] since they generate the embeddings considering the interaction of both sentences, the type of architecture only affects the computational time to obtain the embeddings. Consequently, the architecture type does not affect the size of the computed embeddings, which makes dimension reduction techniques equally interesting and valuable for both architectures.

Therefore, it is noteworthy that the present research pays special attention to the dimension reduction of the embeddings of transformers models trained with siamese architecture because, in Semantic Textual Similarity (STS) tasks, they are a widely used solution that obtains results in a reasonable time and at a feasible computational cost. However, these reduction techniques are equally helpful for other types of architecture.

### Importance of Multilingual Semantics

Semantics has many applications in a wide range of domains and tasks. Recent developments regarding Information Retrieval tasks [34, 51, 52] have demonstrated the potential of combining semantic-aware models along with traditional baseline algorithms (e.g. BM25) [53]. Moreover, the use of semantic-aware models has proven to be an excellent approach to counteract informational disorders (i.e. misinformation, disinformation, malinformation, misleading information, or any other kind of information pollution) [54–57] or to build automated fact-checking approaches [58]. Semantic similarity can be applied to organise data according to text properties, formally an unsupervised thematic analysis [59]. Following the same criterion, the semantic similarity measurement between a sentence and each word can be applied to extract the keywords with the highest semantic content from the sentence [60]. All these applications rely on measuring semantic textual similarity (STS), making STS a crucial task in NLP.

The language bottleneck is a significant limitation of these semantic-aware solutions [61]. Language constitutes one of the most significant barriers to be addressed since a model’s ability to handle multiple languages is essential for its widespread applications. In recent years, in the field of NLP, attention has been paid to multilingual models to achieve diversity in transferring NLP capabilities to low-resource languages [16, 18, 19]. Therefore, in this paper, special attention has been paid to multilingual transformers models, with the immediate goal of covering embedding dimensionality reduction in the semantic textual similarity task in the world’s most widely spoken languages, even though these techniques are relevant for monolingual and multilingual transformers.

Altogether, this work aims to broaden our knowledge of semantic-aware transformer-based models by analysing the impact of different dimensionality reduction techniques on the performance of multilingual siamese transformers on semantic textual similarity multilingual tasks. The results of this study will contribute significantly to understanding how different tuning approaches affect performance on semantic-aware tasks and how dimensional reduction techniques deal with the high-dimensional embeddings computed for the STS task from the recently claimed multilingual point of view.

## Methodology

The main goal of this research lies in providing a deep analysis of the use of different dimensionality reduction techniques to reduce the size of the output embedding of different multilingual transformer models and their impact on performance.

The following subsections describe in detail the techniques explored to reduce embeddings, present the multilingual transformer models included in this study and the different approaches applied to quantify the reduction margin and its effect on the performance.

### Dimensionality Reduction Techniques

As previously mentioned, dimensionality reduction techniques can be grouped according to two non-mutual exclusive criteria. This paper includes a range of types of dimensionality reduction techniques, including linear and nonlinear feature extraction and feature selection techniques. Nevertheless, since the transformers models included in this study are employed in a siamese architecture to determine the degree of similarity between a pair of sentences, they output a pair of non-concatenative embeddings between which the similarity is estimated using the cosine distance. Hence, for each labelled similarity score, there are two non-concatenative embeddings. For this reason, even though we have labelled the data, only unsupervised methods are explored.

The dimensionality reduction techniques explored in this project are:

- **Principal Component Analysis (PCA)**: Principal Component Analysis [45, 46] is a powerful unsupervised linear feature extraction technique that computes a set of orthogonal directions from the covariance matrix that capture most of the variance in the data [62]. This is, it creates new uncorrelated variables that maximise variance, and at the same time, most existing structure in the data is retained. It is also important to note that this research uses a variant of PCA known as Incremental Principal Components Analysis (IPCA) [63]. This variant follows the same basic principles as PCA. However, it is much more memory efficient, as it applies PCA in batches, avoiding storing entire data in memory and allowing PCA to be applied on large datasets.
- **Independent Component Analysis (ICA)** [64]: Independent Component Analysis is an unsupervised feature extraction probabilistic method for learning a linear transformation to find components that are maximally independent between them and non-Gaussian (non-normal), but at the same time, they jointly maximise mutual information with the original feature space.
- **Kernel Principal Components Analysis (KPCA)** [65]: Kernel-based learning method for PCA. It uses kernel functions to construct a nonlinear version of the PCA linear algorithm by first implicitly mapping the data into a nonlinear feature space and then performing linear PCA on the mapped patterns [62]. The kernels considered in this project are the Polynomial, Gaussian RBF, Hyperbolic Tangent (Sigmoid), and Cosine kernels.
- **Variance Threshold**: Unsupervised feature selection approach that removes all features with a variance below a threshold. Indeed, this technique selects a subset of features with large variances, considered more informative, without considering the desired outputs.
- **Uniform Manifold Approximation and Projection for Dimension Reduction (UMAP)**: The authors of UMAP [66] describe it as an algorithm that can be used for unsupervised dimension reduction based on manifold learning techniques and topological data analysis. In short, it first embeds data points in a new nonlinear fuzzy topological representation using neighbour graphs. Secondly, it learns a low-dimensional representation that preserves the complete information of this space, minimising Cross-Entropy. Compared to its counterparts, such as t-SNE, UMAP is fast, scalable, and allows better control of the desired balance between the local and global structure to be preserved. Two main parameters play a vital role in controlling this: (1) the number of sample points that defines a local neighbourhood in the first step, and (2) the minimum distance between embedded points in low-dimensional space to be clustered in the second step. Larger values of the number of neighbours tend to preserve more global information in the manifold as UMAP has to consider more prominent neighbourhoods to embed a point. Likewise, larger minimum distance values prevent UMAP from packing points together and preserving the overall topological structure.

According to the previous preprocessing steps required before dimensionality techniques, it should be noted that PCA and KPCA assume a Gaussian distribution, and the features must be normalised; otherwise, the variances will not be comparable. Therefore, the StandardScaler is applied beforehand. Regarding ICA, non-Gaussian distribution is assumed, and the data is already withened by the algorithm, so no previous preprocessing step is necessary. For the Variance Threshold, the best standardisation method is MinMaxScaler, as it transforms all features to the same scale but does not alter the initial variability. This allows the variance selection threshold set to affect all dimensions equally. Finally, since there are no Gaussian assumptions under UMAP and the cosine distance calculation benefits from scaling the features to a given range, MinMaxScaler is applied before UMAP. A summary of the necessary considerations about the above scaling steps and the characteristics of the dimensionality reduction techniques applied in this project are listed in Table 1.

**Table 1:** Considerations about the previous scaling steps and the characteristics of the different dimensionality reduction techniques applied in this project

	**PCA**	**KPCA**	**ICA**	**Variance Threshold**	**UMAP**
Preprocessor	Standard	Standard		MinMax	MinMax
Scalation	✘	✘	✘	✘	✘
Normalisation	✘	✘			
Unsupervised	✘	✘	✘	✘	✘
Feature Selection				✘	
Feature Extraction	✘	✘	✘		✘
Linear	✘		✘		
Non Linear		✘			✘

**Table 2:** Parameters with non-default values used in the previous scaling steps and the dimensionality reduction techniques applied in this project

**Technique**	**Parameters**
ICA	random_state = 0
	max_iter = 320
	whiten = True
	tol = 5e-4
KPCA	kernels = [sigmoid, polynomial, rbf, cosine]
	eigen_solver = arpack
	copy_X = False
	random_state = 0
Variance Threshold	threshold = [Min, Max, Decile of variance]
UMAP	pre-computed_knn = True
	metric = cosine
	min_dist = 1
	n_neighbors = [5, 10, 50, 100, 125]
	angular_rp_forest = True

Finally, it is worth mentioning that these dimensionality reduction techniques and preprocessing algorithms come from *scikit-learn v1.0.2* [67], except for UMAP, which belongs to *umap-learn* v0.5.2 [66]. For the sake of reproducibility, the different parameters and values used in the experiments are presented in Table 2. Finally, the variance threshold filters for the Variance Threshold technique tested are extracted by previously calculating the variance of each feature (i.e. 768 variances for 768 embedding dimensions), extracting the deciles, and including the maximum and minimum of these variances.

### Multilingual Models

Regarding the models included, we have tried to include the open-source architectures widely used by the NLP community, such as the classic BERT and its robust version of multilingual nature, such as XLM-RoBERTa. The effects of dimensionality reduction and fine-tuning process were explored in the following pre-trained multilingual models extracted from Hugging Face [68]:

- **bert-base-multilingual-cased**: BERT [4] transformer model pre-trained on a large corpus of 104 languages Wikipedia articles using the self-supervised masked language modelling (MLM) objective with $\sim$177M parameters.
- **distilbert-base-multilingual-cased**: Distilled version of the previous model, being on average twice as fast as this model, totalizing $\sim$134M parameters [69].
- **xlm-roberta-base**: Base-sized XLM-RoBERTa [70] model totalizing $\sim$125M parameters. XLM-RoBERTa is RoBERTa model [71], a robust version of BERT, pre-trained on CommonCrawl data containing 100 languages.
- **xlm-roberta-large**: Large-sized XLM-RoBERTa [70] model totalizing $\sim$355M parameters.
- **LaBSE**: Language-agnostic BERT Sentence Embedding [72] model trained for encoding and reducing the cosine distance between translation pairs with a siamese architecture based on BERT, a task related to semantic similarity. It trained over 6 billion translation pairs for 109 languages. The authors also reported that it has zero-shot capabilities, producing decent results for other not seen languages.

Transformer-based models embed textual information into vectors of high dimensionality. The pre-trained multilingual models in this study generate embeddings with 768 dimensions and 1024 in the case of *xlm-roberta-large*. These default dimensions are considered to be reduced since a different number of dimensions from the default would entail losing the knowledge acquired during the self-supervised pre-training phase (e.g. mask word prediction), in which we are interested in studying the effects of the dimension reduction techniques.

**Fig. 1:**
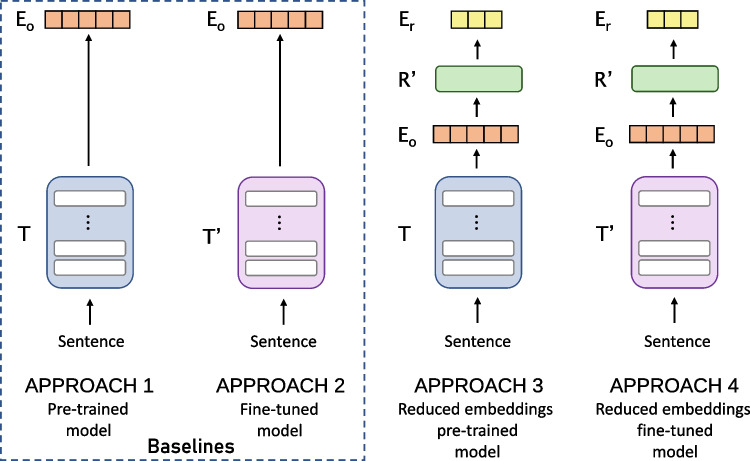
Representation of the approaches followed to evaluate the impact of different dimensionality reduction techniques in multilingual transformers. Where *T* represents the pre-trained transformer model, *T’* the fine-tuned transformer model, *R’* the fitted dimensionality reduction technique, $E_o$ the original output embedding, and $E_r$ the reduced embedding

### Evaluation Approaches

We have followed four approaches to evaluate and quantify the reduction margin and its effect on performance using different methodologies. These four approaches are shown in Fig. 1.

We have included two different baseline approaches, one using the embeddings from the pre-trained version of the five previously mentioned models (i.e. Approach 1) and another using their fine-tuned STS version (i.e. Approach 2). These baselines will allow us to have a standard to examine the dimensionality reduction capability and its effect on STS task performance.

An approach that applies dimensionality reduction to the generated embeddings is used for each baseline. Consequently, Approach 3 applies dimension reduction using Approach 1 as the baseline. Similarly, Approach 4 uses Approach 2 as the baseline.

These four approaches are described as follows:

- **Approach 1 — Pre-trained models**. In the first approach, we employ and directly evaluate the pre-trained models in the mSTSb test split without applying any dimensionality reduction. This approach is used as the baseline for Approach 3.
- **Approach 2 — Fine-tuned models**. In this second approach, the pre-trained models are fine-tuned downstream using the mSTSb train split and evaluated in the mSTSb test split without applying any dimensionality reduction technique. This approach is used as the baseline for Approach 4. The fine-tuning process will be discussed in more detail in the “Transformers Fine-tuning’’ section.
- **Approach 3 — Reduced embeddings from pre-trained models**. In this approach, the embeddings generated by the pre-trained models from Approach 1 in the mSTSb train split are used to fit the different dimension reduction techniques and evaluate them in the mSTSb test split. Thus, an analysis between the results achieved in Approach 1 and Approach 3 will help to understand the impact of dimensionality reduction techniques in the embeddings from pre-trained models.
- **Approach 4 — Reduced embeddings from fine-tuned models**. This approach is equivalent to Approach 3 but uses the fine-tuned models in Approach 2, allowing us to assess the impact of dimensionality reduction techniques in fine-tuned embeddings.

The experimental design of these approaches will be discussed in more detail in the “Baseline Approaches: Approach 1 and Approach 2’’ and “Dimensionality Reduced Techniques Fitting: Approach 3 and Approach 4’’ sections.

## Experimental Setup

### Data

The multilingual extended STS Benchmark (mSTSb) [51] train set is used for fine-tuning the multilingual transformers and fitting the variety of dimensional reduction techniques. This split comprises 16 languages^1^ combined in 31 mono- and cross-lingual tasks with 5, 479 pairs of sentences each one. Likewise, the mSTSb test set is used to evaluate the performance of the models obtained from the different approaches. The mSTSb test set comprises 31 multilingual tasks with 1, 379 pairs of sentences per task.

To evaluate the performance in mSTSb, the sentence embeddings for each pair of sentences are computed and the semantic similarity is measured using the cosine similarity metric. Then, the Spearman correlation coefficient ($\rho$ or $r_s$) is computed between the scores obtained and the gold standard scores, as it is recognised as an official metric used for semantic textual similarity tasks [73, 74].

It is important to note that the mSTSb data variety available for fitting (i.e. train split) totals $+183$ k sentences (i.e. 16 languages with 5, 749 pairs of sentences each). For linear PCA, this dataset is too large to fit in memory. To manage this situation, an Incremental PCA (IPCA) approach [63] is applied. As previously mentioned, IPCA simply fits the PCA in batches, independent of the number of input data samples but still dependent on the input data features.

Similarly, KPCA and UMAP are computationally more expensive than their DIFaddend linear counterparts [62, 65]. For this reason, these dimensionality reduction techniques were fitted using a subset of 10*k* pairs of sentences (i.e. 20*k* sentences), always ensuring the number of data instances is larger than the number of dimensions. To perform this subsampling, the following requirements were taken into account: (1) all 16 languages must be equally represented, giving a total of 625 sentence pairs for each language; (2) all sentences present in the original train split will be present at least once in some language; (3) the representation of the different sentence pairs must be as random as possible. Following these criteria, we perform a sampling based on assigning sentences to a randomly selected language until we reach the maximum number of sampled data. The different sentence pairs are shuffled randomly at each iteration to avoid bias in the order in which the sentences are assigned to the languages. As each language reaches the maximum data, that language is discarded. This ensures a random distribution of samples in each language but includes the full range of sentences in the original train data.

**Fig. 2:**
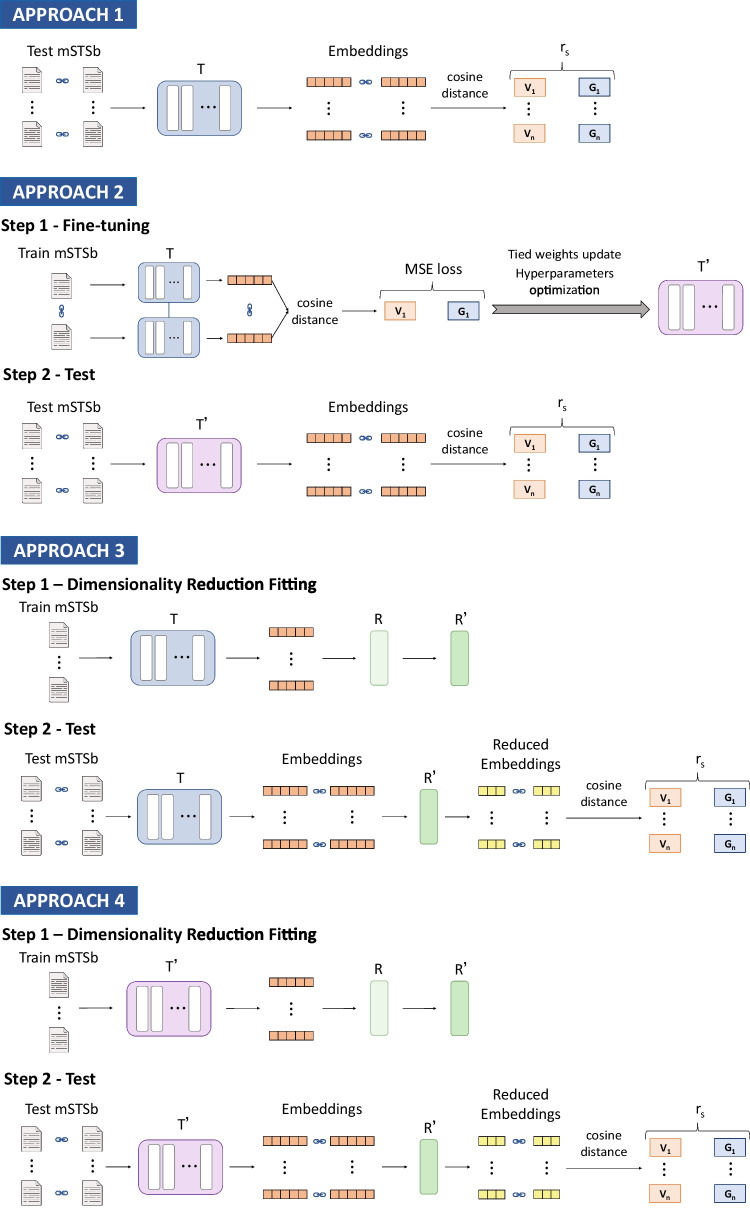
Diagram of the methodology followed for testing the different Approaches on the multilingual STS benchmark. Where *T* represents the pre-trained transformer model, *T’* the fine-tuned transformer model, *R* the dimensionality reduction technique, *R’* the fitted dimensionality reduction technique, $V_i$ the cosine similarity score computed for *i*th sentence pair, $G_i$ the gold standard similarity score for *i*th sentence pair, MSE for Mean Squared Error loss, and $r_s$ the Spearman correlation coefficient

### Computational Resources

Regarding the computational resources used in this study, an Intel(R) Xeon(R) Bronze 3206R CPU at 1.90GHz is used to fit the reduction techniques in Approaches 3 and 4. On the other hand, the pre-computed embeddings used in the approaches and the fine-tuning experiments from Approach 2 are performed using a Quadro RTX 8000 48GB GPU.

### Baseline Approaches: Approach 1 and Approach 2

As shown in Fig. 2, baseline Approaches 1 and 2 share similar experimental designs. Both employ the mSTSb test data split to generate the embeddings of the sentence pairs. As mentioned previously, because they are siamese-trained models, one embedding is computed for each sentence in the sentence pair. Then their similarity value is calculated with the cosine distance, and the Spearman coefficient is computed using the reference values to obtain the baseline performance for each multilingual model. The only difference between Approach 1 and Approach 2 is the version of the model used. In the case of Approach 1, it is the pre-trained version of the model, while in Approach 2 it is the fine-tuned version in the mSTSb task. This fine-tuning process is explained in more detail in the following subsection.

### Transformers Fine-tuning

The fine-tuning process of the models based on the siamese training strategy for Approach 2 was carried out following the methodology described by Reimers et al. [5, 61]. As depicted in the fine-tuning process of Approach 2 in Fig. 2, for each sentence pair from the mSTSb training split, two networks with tied weights from the same transformer model compute the embeddings for each sentence separately. Then the cosine similarity between the two sentence embeddings is calculated. Finally, the mean squared error (MSE) loss between the predicted and the gold similarity scores are used as the objective function to update the tied weights. During training, the following hyperparameters were optimised: number of epochs, scheduler, weight decay, batch size, warmup ratio, and learning rate. The hyperparameter values explored, the required time for fine-tuning, and the results of the experiments can be consulted in Table 8 and Weight and Biases 2.

### Dimensionality Reduced Techniques Fitting: Approach 3 and Approach 4

Figure 2 provides an overview of the process of applying and evaluating dimensional reduction techniques in Approaches 3 and 4. Both approaches follow the same methodology; the only difference is the pre-trained or fine-tuned version of the model used to compute the embeddings for fitting the dimensional reduction technique. The experimental design has two steps, and it is repeated for each number of dimensions explored for each technique and each model. A wide range of the number of dimensions is explored for each dimensionality reduction technique, as shown in the “Results’’ section.

Firstly, a dimensionality reduction technique is fitted through the embeddings computed by a multilingual model using the mSTSb train split. As these are unsupervised techniques (i.e. the gold similarity scores are not included), this training step does not consider the relationship between pairs of sentences. However, it uses individual sentences to fit the technique. In other words, if the train split of mSTSb contains 10*k* pairs of sentences from 16 languages, 20*k* separately sentences are used to fit a dimensional reduction technique.

Finally, the embeddings from paired sentences from the mSTSb test split are reduced with the fitted technique. The cosine similarity distance function is applied to obtain a similarity value, and finally, the Spearman correlation coefficient to the gold similarity scores is computed. In contrast to the previous fitting step, in this second step, the relationship between pairs of sentences is considered as we need to evaluate the techniques on mSTSb task.

### Statistical Comparison

Additionally, to test if the use of reduced embeddings has a significant impact on the performance in comparison to the baseline approaches, we compare the average Spearman correlation coefficient of the five multilingual siamese transformer models (see the “Multilingual Models’’ section) between each pair of baseline and reduced approaches (i.e. Approach 1 vs Approach 3, Approach 2 vs Approach 4). For this purpose, as we are comparing the same set of models in different approaches, the two-tailed paired T-test using a significance level of 0.05 is conducted to test the null hypothesis of identical average Spearman correlation coefficient scores.

**Fig. 3:**
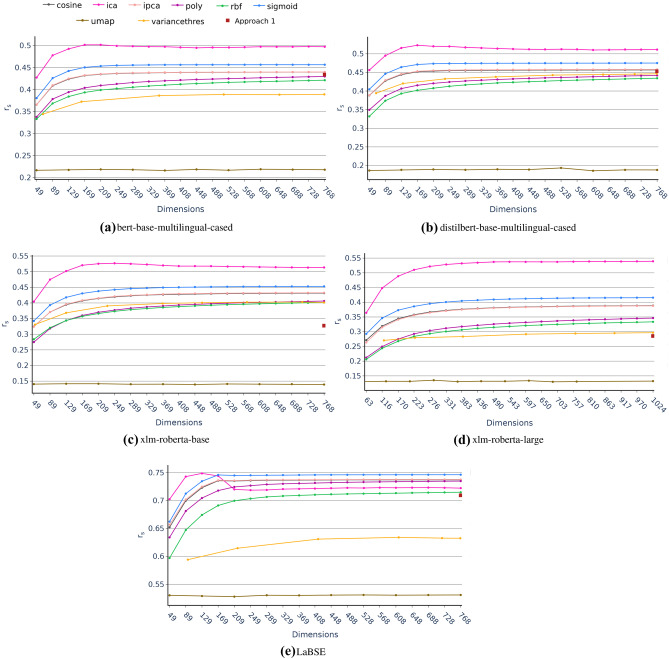
Approach 3 average Spearman $r_s$ correlation coefficient in multilingual tasks from the mSTSb test as a function of the number of dimension for the different dimensionality reduction techniques grouped by model

**Table 3:** Average Spearman $r_s$ correlation coefficient comparison between Approach 1 (Ap. 1) and best dimensional reduction technique in Approach 3 (Ap. 3) for the multilingual Transformers

**Model**	**Ap. 1** $r_s$	**Best Technique**	**Dimensions**	**Ap. 3** $r_s$	**Fitting Time**
bert-base-multilingual-cased	0.4342	ICA	209	0.5019	4 m 16 s
distilbert-base-multilingual-cased	0.4531	ICA	169	0.523	2 m 47 s
xlm-roberta-base	0.3274	ICA	249	0.5269	7 m 51 s
xlm-roberta-large	0.2855	ICA	1024	0.5392	31 m 22 s
LaBSE	0.7096	ICA	129	0.7488	2 m 27 s

**Table 4:** Average Spearman $r_s$ correlation coefficient comparison between Approach 2 (Ap. 2) and best dimensional reduction technique in Approach 4 (Ap. 4) for the multilingual Transformers

**Model**	**Ap. 2** $r_s$	**Best Technique**	**Dimensions**	**Ap. 4** $r_s$	**Fitting Time**
bert-base-multilingual-cased-fine-tuned	0.7045	ICA	568	0.7117	12 m 38 s
distilbert-base-multilingual-cased-fine-tuned	0.6863	VarThres	692	0.6842	2 s
xlm-roberta-base-fine-tuned	0.7470	VarThres	673	0.7495	3 s
xlm-roberta-large-fine-tuned	0.8150	KPCA-sigmoid	1024	0.8176	20 m 6 s
LaBSE-fine-tuned	0.8242	KPCA-sigmoid	768	0.8243	19 m 25 s

## Results

This section aims to summarise the effect of a wide variety of dimensionality reduction techniques on the performance of multilingual siamese transformers by comparing the baseline approaches (i.e. Approaches 1 and 2) with the reduced approaches (i.e. Approaches 3 and 4) for each model independently. It must be noted that this work does not pretend to provide a comparative analysis between the different models presented in the “Multilingual Models’’ section or to identify the best model for this task. In contrast, this work focuses on applying these dimensionality reduction techniques to reduce the dimensionality of the models’ embeddings. Thus, applying different dimensionality reduction techniques does not affect the execution or the memory requirement for running the models. It only diminishes the needed space to store embeddings and increases the speed of computing the cosine similarity between them.

Due to space reasons, average results across the 31 monolingual and cross-lingual tasks are presented instead of a breakdown by language. The average of Spearman correlation coefficients is computed by transforming each correlation coefficient to a Fisher’s *z* value, averaging them, and then back transforming to a correlation coefficient.

### Approach 1 vs Approach 3: Dimensionality Reduction in Pre-trained Embeddings

As can be seen in Fig. 3, for every model, the pre-trained performance on mSTSb (i.e. Approach 1) is improved using different dimensional reduction techniques. These results prove that dimension reduction techniques can somehow adjust the knowledge present in the pre-trained embeddings to the semantic similarity task. This fact becomes even more significant in the case of LaBSE, a model with zero-shot capabilities trained on a task close to semantic similarity, which also greatly benefits from the use of dimension reduction techniques, increasing by 0.4 points the Spearman correlation coefficient (see Fig. 3 and Table 3). For the rest of the models, it is equally remarkable that the dimension reduction techniques improve the pre-training performance, almost doubling the score in the models with the XLM-RoBERTa architecture.

Clearly, the best technique in Approach 3 is ICA. Not only because it obtains the most remarkable improvement in pre-training performance for all models, as shown in Table 3, but also because it is the technique that most quickly and with the fewest dimensions overcomes the pre-trained models of Approach 1 (see Table 5). From the table results, the ICA technique improves the pre-trained Approach 1 performances reducing an average of $91.58\% \pm 2.59\%$ of the initial dimensions retaining $100\%$ of the baseline Approach 1 performance. Remarkably, the two-tailed paired T-test comparing Approach 1 vs Approach 3 using the values for this technique from Table 3 resulted in $p = 0.041$, indicating that the performance improvement is significant when using ICA as a dimensional reduction technique. These findings corroborate the ideas of Raunak et al. [21], who maintained that reduced word embeddings could achieve similar or better performance than original pre-trained embeddings.

**Table 5:** Analysis of the lowest reduced number of dimensions from Approach 3 that improves the result of the baseline Approach 1 for a specific performance threshold retained. For instance, 100% threshold represents that the technique achieves at least the 100% of the baseline approach score

**Model (Ap. 1 Avg** $r_s$)	**Technique**	**Threshold Performance Retained**	**Dimensions (% reduction)**	**Ap. 3 Avg** $r_s$	**Fitting Time**
bert-base-multilingual-cased (0.4342)	IPCA	100%	209 (73%)	0.4251	27 s
	ICA	**100%**	**89 (88%)**	0.4779	1 m 10 s
	poly	95%	249 (68%)	0.4130	49 s
	rbf	95%	448 (42%)	0.4138	1 m 38 s
	sigmoid	100%	129 (83%)	0.4425	40 s
	cosine	100%	209 (73%)	0.4350	36 s
	UMAP	50%	129 (83%)	0.2176	35 s
	VarThres	85%	161(79%)	0.3727	2 s
distilbert-base-multilingual-cased (0.4531)	IPCA	100%	209 (73%)	0.4553	38 s
	ICA	**100%**	**49 (94%)**	0.4564	43 s
	poly	95%	369 (52%)	0.4310	1 m 6 s
	rbf	95%	608 (21%)	0.4305	2 m 13 s
	sigmoid	100%	129 (83%)	0.4642	38 s
	cosine	100%	209 (73%)	0.4537	33 s
	UMAP	40%	49 (94%)	0.3942	18 s
	VarThres	95%	238 (69%)	0.438	2 s
xlm-roberta-base (0.3274)	IPCA	100%	89 (88%)	0.3711	38 s
	ICA	**100%**	**49 (94%)**	0.4043	56 s
	poly	100%	129 (83%)	0.3439	25 s
	rbf	100%	129 (83%)	0.3439	50 s
	sigmoid	100%	49 (94%)	0.3425	29 s
	cosine	100%	89 (88%)	0.3709	15 s
	UMAP	40%	10 (99%)	0.1320	15 s
	VarThres	100%	52 (93%)	0.3310	2 s
xlm-roberta-large (0.2885)	IPCA	100%	116 (89%)	0.3149	1 m 37 s
	ICA	**100%**	** 63 (94%)**	0.3642	1 m 52 s
	poly	100%	223 (78%)	0.2927	45 s
	rbf	100%	276 (73%)	0.2934	1 m 8 s
	sigmoid	100%	63 (94%)	0.2927	32 s
	cosine	100%	116 (89%)	0.3191	25 s
	UMAP	45%	10 (99%)	0.1365	15 s
	VarThres	100%	598 (42%)	0.2917	3 s
LaBSE (0.7096)	IPCA	100%	129 (83%)	0.7251	37 s
	ICA	**100%**	** 89 (88%)**	0.7431	1 m 35 s
	poly	100%	169 (78%)	0.7181	34 s
	rbf	100%	408 (47%)	0.7106	1 m 35 s
	sigmoid	100%	89 (88%)	0.7127	34 s
	cosine	100%	129 (83%)	0.7232	21 s
	UMAP	70%	10 (99%)	0.5026	33 s
	VarThres	85%	217 (72%)	0.6148	2 s

**Table 6:** Analysis of the lowest reduced number of dimensions from Approach 4 that improves the result of the baseline Approach 2 for a specific performance threshold retained. For instance, 100% threshold represents that the technique achieves at least the 100% of the baseline approach score

**Model** **(Ap. 2** **Avg** $r_s$)	**Technique**	**Threshold** **Performance** **Retained**	**Dimensions (% reduction)**	**Ap. 4** **Avg** $r_s$	**Fitting Time**
bert-base-multilingual-cased-fine-tuned (0.7045)	IPCA	95%	49 (94%)	0.6710	34 s
	ICA	**100%**	**169 (78%)**	0.7047	3 m 37 s
	poly	95%	129 (83%)	0.6716	31 s
	rbf	95%	169 (78%)	0.6738	54 s
	sigmoid	100%	329 (57%)	0.7048	1 m 34 s
	cosine	95%	49 (94%)	0.6707	11 s
	UMAP	70%	10 (99%)	0.5398	32 s
	VarThres	100%	393 (53%)	0.7046	2 s
distilbert-base-multilingual-cased-fine-tuned (0.6863)	IPCA	**95%**	**49 (94%)**	0.6533	35 s
	ICA	**95%**	**49 (94%)**	0.6556	56 s
	poly	95%	129 (83%)	0.6542	30 s
	rbf	95%	129 (83%)	0.6520	49 s
	sigmoid	**95%**	**49 (94%)**	0.6601	29 s
	cosine	95%	89 (88%)	0.6631	16 s
	UMAP	75%	10 (99%)	0.5189	25 s
	VarThres	95%	66 (91%)	0.6620	2 s
xlm-roberta-base-fine-tuned (0.7470)	IPCA	95%	49 (94%)	0.7198	36 s
	ICA	95%	49 (94%)	0.7208	59 s
	poly	95%	89 (88%)	0.7112	25 s
	rbf	95%	129 (83%)	0.7134	49 s
	sigmoid	**100%**	**289 (62%)**	0.7472	1 m 45 s
	cosine	95%	49 (94%)	0.7195	11 s
	UMAP	75%	10 (99%)	0.5724	31 s
	VarThres	100%	411 (46%)	0.7491	3 s
xlm-roberta-large-fine-tuned (0.8150)	IPCA	95%	63 (94%)	0.7910	51 s
	ICA	95%	63 (94%)	0.7950	1 m 16 s
	poly	95%	63 (94%)	0.7774	23 s
	rbf	95%	63 (94%)	0.7760	42 s
	sigmoid	**100%**	**223 (78%)**	0.8151	50 s
	cosine	95%	63 (94%)	0.7916	13 s
	UMAP	80%	10 (99%)	0.6584	38 s
	VarThres	95%	95 (91%)	0.7936	3 s
LaBSE-fine-tuned (0.8242)	IPCA	95%	89 (88%)	0.8014	34 s
	ICA	95%	89 (88%)	0.7986	2 m 3 s
	poly	95%	89 (88%)	0.7898	25 s
	rbf	95%	129 (83%)	0.7932	47 s
	sigmoid	**100%**	**728 (5%)**	0.8243	7 m 11 s
	cosine	95%	89 (88%)	0.8001	21 s
	UMAP	80%	23 (97%)	0.6640	35 s
	VarThres	95%	227 (70%)	0.7964	2 s

The most likely explanation for these results is the difference in the objective of ICA from the other feature extraction techniques. Even though they all transform the initial space through combinations of dimensions into a new space, the ICA technique is based on optimising mutual information [64, 75]. It tries to find a space where the new features (latent variables) are as independent as possible from each other but as dependent as possible on the initial space. Therefore, in the case of ICA, unlike other techniques such as PCA or KPCA, a higher number of components does not necessarily mean an increase in the information retained or an improvement in the result (as can be seen in Fig. 3 and clearly in Fig. 3 where there is a decrease from 169 dimensions onwards). This would explain why a low number of dimensions would outperform Approach 1.

Likewise, the fact that it is the technique that achieves the best results in Approach 3 in all models could be due to the assumptions and characteristics of both the ICA and the pre-trained embeddings. First, the pre-trained embeddings probably include non-relevant and noisy variables as these embeddings are not adjusted to the STS task. Secondly, since ICA is a technique in which the original variables are related linearly to the latent variables but for which the latent distribution is non-Gaussian, the noise present in pre-trained embeddings agnostic of the STS task could be managed appropriately.

Interestingly, these results also emphasise that the issue of non-Gaussianity is more relevant than the nonlinearity issue. Non-Gaussianity would be more important than how the initial variables are combined, as the ICA technique outperforms linear PCA and nonlinear KPCA. This is in good agreement with other studies comparing the performance of PCA and ICA as a method for feature extraction in visual object recognition tasks [76, 77].

Additionally, the presence of noisy variables in the pre-trained embeddings would also be corroborated by the low scores obtained from the Variance Threshold feature selection technique, which entirely depends on the original variables and cannot manage these noisy distributions.

Consequently, ICA shows excellent properties for obtaining compacted embeddings versions of pre-trained models with a significant decrease of dimensions that improve the result in the task of semantic similarity at a multilingual level.

For all these reasons, we can understand unsupervised dimensionality reduction, specially ICA, as a method of fitting pre-trained models for downstream tasks. As it will be seen in the next section and as might be expected, this unsupervised dimensionality reduction downstream fitting is not comparable to a supervised fitting such as the fine-tuning of Approach 2. However, downstream fitting by unsupervised dimensionality reduction techniques may present interesting advantages such as the fact that being unsupervised is task agnostic resulting in models with higher generalizability and with a lower number of dimensions. Also, these dimensionality reduction techniques do not require GPUs and apply a more interpretable methodology than a Deep Learning model fine-tuning such as transformers.

**Fig. 4:**
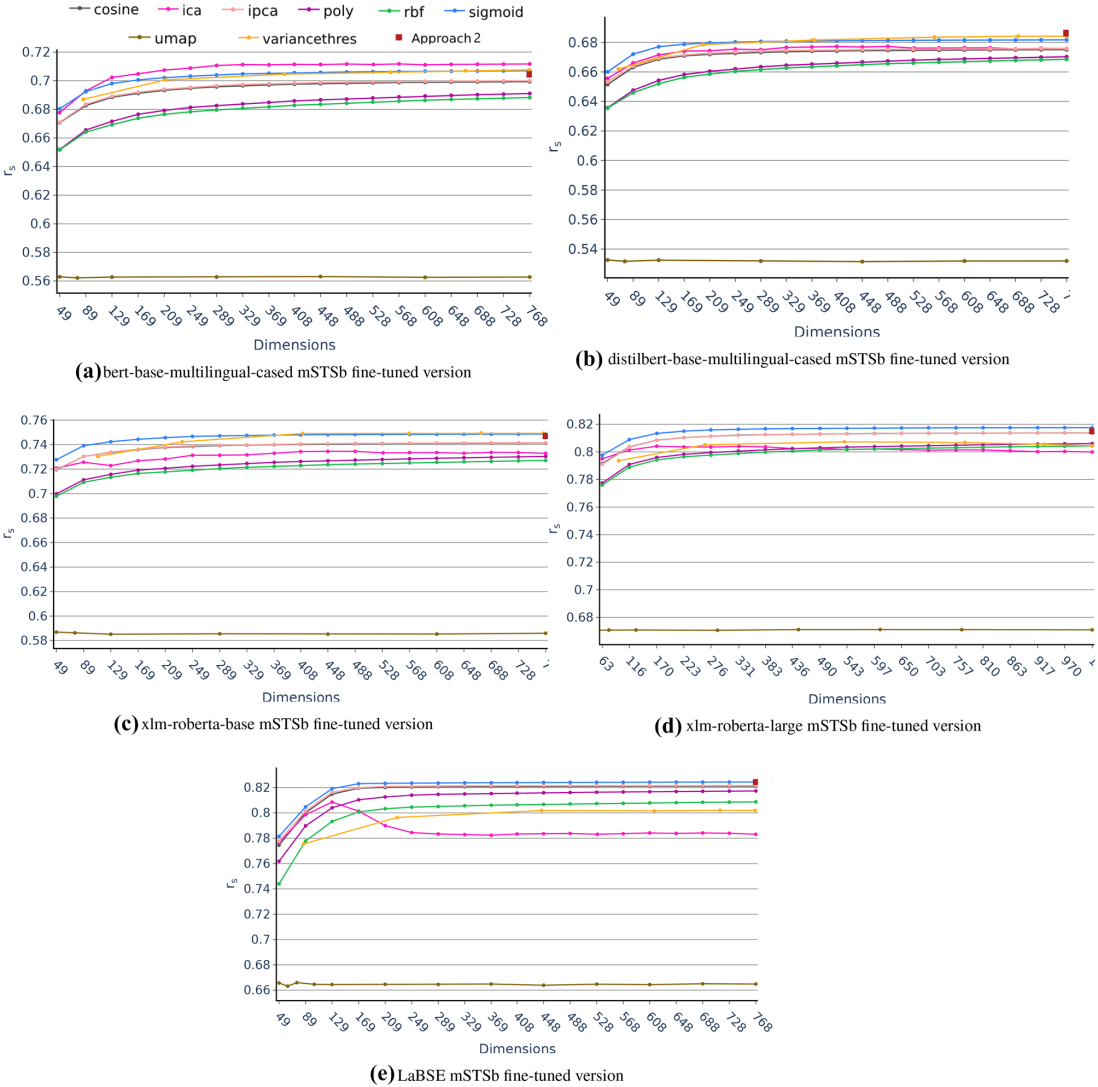
Approach 4 average Spearman $r_s$ correlation coefficient in multilingual tasks from the mSTSb test as a function of the number of dimensions for the different dimensionality reduction techniques grouped by model

### Approach 2 vs Approach 4: Dimensionality Reduction in Fine-tuned Embeddings

Although it was stated at the beginning of this section that model comparison is not an objective of the paper, different versions of the same architecture have been included for a more comprehensive evaluation of the effects of dimensionality reduction techniques (i.e. *xlm-roberta-base* and *xlm-roberta-large*, *bert-base-multilingual-cased* and *distilbert-base-multilingual-cased*) have been included in the study. Based on the complexity and learning potential of the models, one would expect the *xlm-roberta-large* model to perform better than the *xlm-roberta-base *model. Similarly, the *bert-base-multilingual-cased* model would be expected to be superior to the *distilbert-base-multilingual-cased* model. In Approach 1, however, the opposite is true. Only when fine-tuning occurs in the task (Approaches 2 and 4) is it observed how the performance of the models is in line with the expected complexity (see Table 4). These results provide wider support for the importance of supervised fine-tuning.

Similarly, fine-tuning also alters the impact of dimensionality reduction techniques on the results of multilingual models. Compared to Approach 3, when fine-tuning, the feature selection techniques and nonlinearity become more important, ICA becomes less critical, and the minimum number of dimensions that outperform the baseline approach increases.

As can be seen in Fig. 4 and Table 4, the promotion of Variance Threshold feature selection as one of the best techniques for some models in Approach 4 could be attributed to the fact that fine-tuning adjust the embeddings to the task, reducing the presence of noisy variable and taking advantage of the variable selection process. This would be in line with the results obtained in Approach 3, where feature extraction techniques more adequately handled the presence of unadjusted variables. This further supports the argument that they can reduce dimensions and generate new feature representations to help improve performance on learning problems.

**Table 7:** Time required to fit the different dimension reduction techniques in the mSTSb task. Owing to the fact that a wide range of dimensions is explored, the fitting time for the minimum, in-between and maximum number of dimensions are reported for each model and technique. The measurements are performed on an Intel(R) Xeon(R) Bronze 3206R CPU at 1.90GHz

**Model**	**Technique**	**Time Min Dimension**	**Time In-between Dimension**	**Time Max Dimension**
bert-base-multilingual-cased	IPCA	27 s (10)	29 s (448)	30 s (768)
	ICA	53 s (10)	8 m 43 s (448)	52 m 48 s (768)
	poly	18 s (10)	1 m 26 s (448)	2 m 17 s (768)
	rbf	36 s (10)	1 m 38 s (448)	3 m 29 s (768)
	sigmoid	24 s (10)	2 m 15 s (448)	5 m 53 s (768)
	cosine	8 s (10)	55 s (408)	1 m 24 s (768)
	UMAP	20 s (10)	1 m 30 s (448)	3 m 29 s (768)
	VarThres	2 s (65)	2 s (516)	2 s (767)
distilbert-base-multilingual-cased	IPCA	36 s (10)	40 s (448)	44 s (768)
	ICA	29 s (10)	8 m 30 s (448)	29 m 31 s (768)
	poly	16 s (10)	1 m 24 s (448)	2 m 35 s (768)
	rbf	35 s (10)	1 m 44 s (448)	2 m 57 s (768)
	sigmoid	23 s (10)	3 m 58 s (448)	15 m 40 s (768)
	cosine	8 s (10)	51 s (448)	1 m 51 s (768)
	UMAP	13 s (10)	1 m 38 s (448)	4 m 19 s (768)
	VarThres	2 s (66)	2 s (507)	2 s (767)
xlm-roberta-base	IPCA	35 s (10)	35 s (448)	40 s (768)
	ICA	56 s (10)	9 m 54 s (448)	21 m 9 s (768)
	poly	16 s (10)	1 m 2 s (448)	2 m 12 s (768)
	rbf	33 s (10)	1 m 38 s (448)	2 m 2 s (768)
	sigmoid	23 s (10)	1 m 43 s (448)	3 m 26 s (768)
	cosine	7 s (10)	56 s (448)	1 m 36 s (768)
	UMAP	15 s (10)	2 m (448s)	4 m 32 s (768)
	VarThres	2 s (1)	2 s (448)	2 s (768)
xlm-roberta-large	IPCA	54 s (10)	54 s (490)	1 m 46 s (1024)
	ICA	57 s (10)	23 m 13 s (490)	32 m 15 s (1024)
	poly	16 s (10)	1 m 24 s (490)	3 m 35 s (1024)
	rbf	35 s (10)	1 m 30 s (490)	4 m 49 s (1024)
	sigmoid	23 s (10)	2 m 58 s (490)	8 m 28 s (1024)
	cosine	9 s (10)	1 m 38 s (490)	2 m 41 s (1024)
	UMAP	15 s (10)	1 m 57 s (490)	7 m 24 s (1024)
	VarThres	3 s (10)	3 s (598)	3 s (1024)
LaBSE	IPCA	35 s (10)	36 s (448)	39 s (768)
	ICA	36 s (10)	12 m 55 s (448)	52 m 39 s (768)
	poly	17 s (10)	1 m 24 s (448)	2 m 35 s (768)
	rbf	35 s (10)	1 m 41 s (448)	2 m 50 s (768)
	sigmoid	24 s (10)	4 m 25 s (448)	19 m 37 s (768)
	cosine	8 s (10)	55 s (448)	1 m 44 s (768)
	UMAP	33 s (10)	2 m 11 s (448)	4 m 18 s (768)
	VarThres	2 s (94)	2 s (615)	2 s (767)

**Table 8:** Time required to fine-tune the different models in the mSTSb task. The fine-tuning process is carried out in Quadro-RTX 8000 GPU. The best hyperparameters configuration considered for each model is also reported

**Model**	**Time**	**Hyperparameters**
bert-base-multilingual-cased-fine-tuned	39 m 18 s	bach_size: 32
		lr: 2e-5
		epochs: 2
		scheduler: warmuplinear
		warmup_ratio: 0.2
		wieght_decay: 0.2
distilbert-base-multilingual-cased-fine-tuned	15 m 31 s	bach_size: 64
		lr: 2e-5
		epochs: 2
		scheduler: warmuplinear
		warmup_ratio: 0.3
		wieght_decay: 0.7
xlm-roberta-base-fine-tuned	27 m 49 s	bach_size: 64
		lr: 5e-5
		epochs: 2
		scheduler: warmuplinear_hard_restarts
		warmup_ratio: 0.1
		wieght_decay: 0.5
xlm-roberta-large-fine-tuned	1 h 2 m 32 s	bach_size: 64
		lr: 1e-5
		epochs: 2
		scheduler: warmupcosine
		warmup_ratio: 0.2
		wieght_decay: 0
LaBSE-fine-tuned	59 m 39 s	bach_size: 32
		lr: 3e-6
		epochs: 2
		scheduler: warmupcosine
		warmup_ratio: 0.1
		wieght_decay: 0.5

Furthermore, Table 6 shows the lack of ICA dominance and the emergence of the KPCA-sigmoid technique as the method with fewer dimensions improves the baseline Approach 2 ($59.32\% \pm 29.92\%$ reduced dimensions retaining $99.00\% \pm 2.00\%$ baseline performance). It reveals that managing the non-Gaussianity issue is less relevant than the nonlinearity issue after fine-tuning. The fine-tuning process also impacts the reduction capabilities of the dimensionality reduction techniques since considering the technique that retains the maximum performance with the lowest dimensions for each model in Table 6 shows that the initial dimensions from the baseline Approach 2 are reduced by an average of $54.65\% \pm 32.20\%$. Although this average reduction is lower than the achieved earlier in the comparison of Approach 3 with the baseline Approach 1, it is still remarkable that even after fine-tuning, the multilingual performance can be exceeded with half of the dimensions. Finally, the two-tailed paired T-test comparing Approach 2 vs Approach 4 using the values from Table 4 resulted in $p = 0.255$, revealing no significant difference in performance using these dimensional reduction techniques.

### Execution Time Comparison

Although it is out of the main scope of the paper, this subsection compares the different dimension reduction techniques regarding the execution time required to fit each technique. As mentioned in the “Data’’ section, it is fundamental to note that KPCA and UMAP were fitted using a subset of 10*k* pairs of sentences due to their computational cost, which is more expensive than their linear counterparts. Therefore, for fair comparisons, IPCA, ICA, and variation threshold techniques are compared on the one hand and KPCA and UMAP on the other.

Finally, we analyse the reduction of computational time by comparing the fitting time of these techniques in Approach 3 and 4 concerning the fine-tuning process performed in Approach 2, following the experimental design described in the “Experimental Setup’’ section.

#### Dimensionality Reduction Techniques Comparison: IPCA, ICA and Variance Threshold

Considering the wide range of dimensions explored for each model, we analyse the fitting time for the minimum, the in-between, and the maximum number of dimensions technique in the mSTSb train split to compare the different techniques in execution time for Approaches 3 and 4. The results of these tests and the dimensions associated with these times are presented in Table 7. It should be noted that the number of dimensions shown in this table for the variance threshold technique differs from the other techniques since the number of reduced dimensions depends on the number of dimensions that exceed the variance threshold explored.

Table 7, shows that the technique that requires the longest fitting time of IPCA, ICA and variance threshold is ICA, while the variance threshold is the one that requires the shortest time.

Regarding computational complexity, IPCA has constant memory complexity $O(bd^2)$ [67], while the ICA algorithm is $O(2md(d+1)n)$ [78], where *n* is the number of samples of dimensions *d*, *b* is the batch size for the IPCA algorithm, and *m* is the number of iterations of the ICA algorithm. We can observe that when IPCA and ICA are compared for the same number of dimensions and number of samples, the computational burden of the ICA technique is the number of iterations (*m*) since they critically increase execution time. As stated in [78], this effect is more noticeable as the dimension increases, making the use of ICA computationally unfeasible when the number of dimensions is moderate or high. Our results support these findings, as the ICA’s fitting time explodes when the number of dimensions is high (see Table 7). On the other hand, the IPCA execution time remains practically constant because the batches avoid attempting to load all the data into memory at the same time.

Finally, as expected, the feature selection technique is the least time-consuming since it only requires computing the variance for each dimension and filtering it according to a selected variance threshold.

Consequently, there is a trade-off between the number of dimensions to be reduced, the fitting time, and the percentage of performance to be retained. We would recommend that for the case of pre-trained embedding reduction, the ICA technique should be the priority if a low number of dimensions is explored since it is the best at retaining and improving the initial performance at a feasible cost. However, suppose the target number of dimensions to be reduced is medium or high. In that case, we recommend the IPCA technique since it can retain and improve performance with a constant computational cost. Finally, suppose that the execution time is clearly prioritised. In that case, we recommend the variance threshold feature selection technique, even if the number of dimensions is reduced and the performance retained is not the best, as this technique cannot handle the noise in pre-trained features not adjusted for a downstream task.

#### Dimensionality Reduction Techniques Comparison: KPCA and UMAP

Regarding the KPCA technique, using a kernel function before performing linear PCA increases the execution time. These kernel functions map the data into a nonlinear feature space, so the execution time varies depending on the kernel type. From the results shown in Table 7, the sigmoid kernel requires the most execution time and the cosine kernel the least. Despite these previous results, the selection of the kernel type depends on each case, and different kernels should be explored, as previous studies suggest [62, 65].

Finally, it can be seen from the results shown in Table 7 that the execution time of UMAP is similar to KPCA. Nevertheless, several considerations must be taken into account in the case of the UMAP technique. As discussed in the “Dimensionality reduction Techniques’’ section, the number of neighbours parameter is proportional to the amount of global information retained. As shown in Table 2, different values of the number of neighbours are explored for each model. The number of neighbours that provided the best results were 10, 5, 5, 5 and 125 for each model in the same order as the models shown in Table 7. The value of this parameter determines the execution time of the technique; increasing the time, the greater the number of neighbours to consider for embedding a sentence. For this reason, the degree of similarity between KPCA and UMAP run times varies from one model to another. Similarly, although the table only includes the execution times for the best UMAP parameter values found for each model, the choice of these parameters requires a prior exploration process that can be very long, depending on how intensive the search for the best parameter values is. Hence, this technique has an additional cost to consider.

#### Fitting Dimensionality Reduction Technique and Fine-tuning Comparison

Another point to discuss is comparing the execution times required by dimension reduction techniques with the fine-tuning process. It should be noted that the fine-tuning times shown in Table 8 do not include the time required for hyperparameter optimization, which, depending on how extensive the search is, can extend the time required to obtain a fine-tuned model. Therefore, the time shown refers to the execution time needed to fine-tune each model once the optimal hyperparameters are found.

In practical application, the time required for fitting a dimensionality reduction technique on pre-trained embeddings instead of fine-tuning the model downstream is what matters. For this purpose, we use the fine-tuning process execution times shown in Table 8 compared to Table 5, which shows the fitting time of the dimension reduction techniques of Approach 3 that improves the result of the baseline Approach 1.

For space reasons, even though Table 5 shows a breakdown of the performance for each technique, the technique used to compare the fine-tuning of each model is the technique that, with the lowest number of dimensions, retains the highest possible performance (highlighted in bold in Table 5). As stated previously, the best technique for all models is ICA. This technique requires a fitting time of an average of $96.68\% \pm 0.68\%$ faster than the fine-tuning process on all models.

Taken together, we can see the advantage in execution time of using reduction techniques in Approach 3 with respect to the fine-tuning process carried out in Approach 2. Remarkably, it is exciting to point out the potential of ICA as an alternative to the fine-tuning process. Although it has previously been proven that fine-tuning achieves better performance than fitting dimension reduction techniques, this technique does not require GPU. This technique reduces the execution time by more than 96%. This fact, together with the ability to improve the performance of pre-trained embeddings and the generalisation capability of this unsupervised technique, reveals the potential of this technique as an alternative to fine-tuning.

### UMAP: a Case of Study

In this section, we pay special attention to the recently proposed UMAP technique [66]. The case of UMAP shows that for the STS task, it is not a suitable technique to reduce the dimensionality of the embeddings since it is the one that retains the lowest percentage of the baseline performances in both pre-trained and fine-tuned embeddings (see Tables 5 and 6). Considering this fact, it is interesting to note that the potential of this technique resides in the fact that it quickly saturates, i.e. the maximum retained performance is reached with a small number of dimensions in Approach 3 with an average of $94.65\% \pm 6.07\%$ of reduced initial dimensions retaining $49.00\% \pm 11.14\%$ performance concerning the reference Approach 1, and more notably in Approach 4 with an average of $98.42\% \pm 0.72\%$ of reduced initial dimensions retaining $76.00\% \pm 3.74\%$ performance for the reference Approach 2.

This saturation behaviour can be attributed to the demonstrated functionality of UMAP for generating visualisations by reducing high-dimensional data to 2- or 3-dimensions. As reported in other works [79, 80], the most significant potential of this technique lies precisely in visualisation, where this saturation capability is exploited. UMAP is a nonlinear graph-based method for dimensionality reduction that is not meant to extract features as methods like PCA do [66]. Instead, its primary goal is to represent the input data as a high-dimensional graph and then reconstruct it in a lower-dimensional space while retaining structure. These characteristics explain the lack of good results for the UMAP technique in the mSTSb task in Approach 3, where feature extraction has proven to help manage the noisy pre-trained features. Furthermore, this would explain why we observe UMAP saturation with fewer dimensions in Approach 4, as the embeddings are already fine-tuned, and UMAP can exploit its potential to preserve information.

Previously, it has been proven that its fitting requires an execution time similar to other techniques such as KPCA. It is also important to note that its computational cost limits the total number of data instances to be used for fitting since the first step is expensive. It requires computing a graphical representation of the dataset and then learning an embedding for that graph.

Despite the previous conclusion, UMAP is time-consuming. This technique highly depends on the parameters used, and many parameters can be explored. Additionally, the slow process of embedding new data in a fitted UMAP must also be considered. UMAP does not build a function that directly maps high-dimensional points down to a reduced space. Instead, it first computes a graph representing the whole data and then learns an embedding for it. Thus, embedding a new point requires calculating its nearest neighbours from the fitting data and embedded in the learnt graph. Again, this process execution time increases with the number of neighbours value selected when. Therefore, UMAP can transform new data, albeit more slowly than other techniques that allow this.

### Previous Works Comparison

Finally, we discuss our results concerning previous related work in dimensionality reduction of pre-computed embeddings. Before this discussion proceeds, it is essential to remark that our work differs from previous work in the literature as we explore a more comprehensive range of unsupervised dimension reduction techniques, evaluating them on pre-trained and fine-tuned pre-computed embeddings from state-of-the-art multilingual contextual-based transformer models in the Semantic Textual Similarity (STS) task.

Our experimental results are in agreement with Raunak et al. [21, 22], Singh et al. [27] and Thirumoorthy and Muneeswaran [37], which corroborates the hypothesis that reduction in pre-trained embeddings can maintain or improve the performance of the original embeddings. Similarly, our results are consistent with those obtained by Truşcă et al. [23] and Shimomoto et al. [47]. The authors employ PCA as the only unsupervised technique. We have confirmed that this feature extraction technique has great potential to reduce the dimensions of pre-trained embeddings. Our results further reveal that incremental PCA (IPCA) is also suitable for embedding reduction. Besides, we found that for embeddings already fine-tuned to the downstream task, using the KPCA, the nonlinear version of PCA, is much more helpful for preserving performance and reducing dimensions.

Regarding execution time, our results are similar to Saeed et al. [17]. These authors combined the unsupervised PCA technique and the supervised LDA technique with classical NLP techniques based on N-grams and classical ML models (Decision Trees, Logistic Regression, or Naive Bayes) for sentiment classification of monolingual Arabic texts. The authors reported an improvement in results over previous work by reducing dimensions by up to 93% with a 97% shorter run time.

Even though the NLP task addressed in the present study is STS from a multilingual perspective (including Arabic) and not sentiment classification, our findings align with these previous results. The ICA technique reduces an average of $91.58\% \pm 2.59\%$ of the initial dimensions, retaining 100% of the baseline Approach 1 performance, requiring a fitting time of $96.68\% \pm 0.68\%$ faster than the fine-tuning process.

Given these points, we can safely conclude that our results are in line with previous work extending their findings to state-of-the-art contextual-based models from a multilingual approach. This paper provides new insights into dimensionality reduction techniques for a space- and time-efficient data representation.

## Conclusion

In this investigation, the goal was to assess the impact of a variety of dimensionality reduction techniques on the performance of pre-computed multilingual siamese fashion transformers embeddings on semantic textual similarity tasks from mSTSb, to expand our knowledge of semantic-aware transformer-based models. To this end, two different baseline approaches are reduced (i.e. Approach 1 and Approach 2), one using the pre-trained version of the models and the second further fine-tuning them on the downstream STS task. Particular attention is paid to analysing which techniques best and with the fewest dimensions improve the performance of the baseline approaches.

From the research, it is possible to conclude that dimensionality reduction techniques can help reduce the number of dimensions of the embeddings while improving the results if using pre-trained embeddings from Approach 1 and preserving the performance when using fine-tuned embeddings from Approach 2. Nevertheless, the dimensionality reduction is more considerable in the pre-trained version with an average of $91.58\% \pm 2.59\%$ compared to the average of $54.65\% \pm 32.20\%$ of the fine-tuned version. Special attention is given to ICA in the pre-trained scenario, which adequately managed the noisy variables present in not adjusted embeddings. This technique also proved to be a reasonable alternative to fit the models in the downstream task in an unsupervised way, leading to a generalised adjusted version of the models with downstream multitasking capabilities. Nevertheless, it has also been proved that this unsupervised fitting is not comparable to supervised fine-tuning. On the other hand, the fine-tuned scenario revealed the relevance of feature selection techniques and the significance of nonlinear KPCA techniques for dimensionality reduction.

Additionally, although our execution time experiments reveal that ICA is the most time-consuming technique among the dimension reduction techniques, reducing pre-trained embeddings (Approach 3) by gaining generalisation power with this unsupervised technique is still faster than performing downstream fine-tuning (Approach 2). Moreover, ICA does not require GPUs for fitting, reinforcing its potential as an alternative to fine-tuning. Overall, a good trade-off between performance against available computational resources, execution time, and the number of dimensions to be reduced must be considered when choosing the approach to follow.

The results of our experiments are consistent with previous results from the literature, corroborating the hypothesis that reduction in embeddings can maintain or improve the performance of the original embeddings by extending their evaluation to state-of-the-art contextual-based models from a multilingual approach. In this way, we can establish that dimensionality reduction techniques could also be leveraged for contextualised embeddings.

To our knowledge, this is the first study to investigate the effect of dimensionality reduction techniques and transformers models in a multilingual semantic-awareness scenario. This study analyses alternatives to the storage limitation we are about to face if the current trends of using large datasets and the growth rate of storage utilisation persist. Based on the promising findings presented in this article, continued research into the impact of dimensionality reduction techniques in other highly demanded NLP tasks appears to be totally justified. Furthermore, in future research, we intend to focus on testing the reduced models presented in this work in real-world applications. Besides, we hope to carry out further experimental investigation, including other dimensionality reduction approaches, such as creating a distilled version of pre-trained models or exploring the novel feature extraction and feature selection methods proposed in [27, 37].

As stated previously, the findings presented in this study of multilingual semantic similarity are of direct practical applicability. Combining dimensionality reduction techniques with transformer models could also help reduce the embedding size and make ensemble approaches possible. Finally, further studies about the multitasking generalisation capabilities of ICA for pre-trained models are still required.

## Data Availability

The Multilingual Semantic Textual Similarity Benchmark (mSTSb) used for the purpose of this article can be found in https://github.com/Huertas97/Multilingual-STSB.

## Footnotes

1 ar, cs, de, en, es, fr, hi, it, ja, nl, pl, pt, ru, tr, zh-CN, zh-TW

2 https://wandb.ai/huertas_97/PaperDimRed
